# Influence of Remimazolam and Propofol on Intraoperative Motor Evoked Potentials During Spinal Surgery: A Randomized Crossover Trial

**DOI:** 10.3390/jcm14155491

**Published:** 2025-08-04

**Authors:** Bo Rim Kim, Hye-Bin Kim, Moo Soo Kim, Byung Gun Lim, Seok Kyeong Oh

**Affiliations:** 1 Department of Anesthesiology and Pain Medicine, Asan Medical Center, University of Ulsan College of Medicine, Seoul 05505, Republic of Korea; petitbelle22@naver.com; 2 Department of Anesthesiology and Pain Medicine, Korea University Guro Hospital, Korea University College of Medicine, Seoul 08308, Republic of Korea; aneshbkim@korea.ac.kr (H.-B.K.); ottawa2525@gmail.com (M.S.K.); bglim9205@korea.ac.kr (B.G.L.)

**Keywords:** remimazolam, motor evoked potentials, propofol, crossover study, neurophysiological monitoring, anesthesia

## Abstract

**Background/Objectives:** Total intravenous anesthesia (TIVA) typically combines propofol and remifentanil. Remifentanil exerts minimal influence on motor evoked potential (MEP), whereas propofol partially reduces MEP amplitude. Remimazolam, a novel agent, is a component of TIVA. However, evidence of remimazolam on MEP is limited. We aimed to compare the effects of propofol and remimazolam, combined with remifentanil, on relative MEP depression. **Methods:** Using a crossover design, 18 patients undergoing spine surgery were randomly assigned to receive either propofol or remimazolam as the first agent. In the propofol first sequence, anesthesia was induced and maintained with propofol, which was then switched to remimazolam 60 min after surgery. In the remimazolam first sequence, remimazolam was used first and then switched to propofol. The primary outcomes measured were the MEP amplitude and latency. **Results:** MEP amplitude and latency during propofol and remimazolam infusions were as follows: amplitude (mean (SD); 635.3 (399.1) vs. 738.4 (480.4) μV, *p* = 0.047) and latency (median [IQR]; 22.4 [20.3–24.6] vs. 21.4 [19.6–23.5] ms, *p* = 0.070), indicating propofol caused greater depression in amplitude than remimazolam. However, an incident of severe body movement disrupting surgery occurred under remimazolam anesthesia in a young, healthy male patient, although bispectral index remained below 60. This suggests that remimazolam, at hypnotic levels similar to propofol, may result in reduced akinesia in major surgeries, such as spinal surgery, when neuromuscular blockade is not employed. **Conclusions:** Remimazolam demonstrated comparable or superior effects to propofol on MEP latency and amplitude when combined with remifentanil during spinal surgery, rendering it a potential alternative to propofol for MEP monitoring.

## 1. Introduction

Intraoperative neurophysiological monitoring, including motor evoked potentials (MEPs), is widely employed to prevent inadvertent injury during spinal surgery [1]. By evaluating the amplitude and latency of MEPs, clinicians can monitor the functional integrity of motor pathways in real time and respond immediately to emerging neural risks [1,2]. Among anesthetic agents, volatile inhalational anesthetics such as sevoflurane and desflurane are known to significantly depress MEP signals, particularly at concentrations exceeding 0.5 minimum alveolar concentration. Therefore, total intravenous anesthesia (TIVA), using agents with a lesser effect on MEPs, such as propofol and remifentanil, is commonly preferred for these procedures [3].

Remifentanil, an ultra-short-acting synthetic opioid, has minimal impact on MEPs and is routinely used in conjunction with hypnotics during neuromonitoring [3,4,5]. Propofol, a widely used hypnotic for TIVA, has been shown to partially reduce MEP amplitude [6]. Nevertheless, its titratability and rapid metabolism have made it the standard hypnotic agent in surgeries requiring MEP monitoring [6,7].

Remimazolam is a novel short-acting benzodiazepine, designed to enable rapid onset and offset due to its metabolism by carboxylesterase-1. Its short context-sensitive half-life and growing evidence supporting stable hemodynamics have increased its use in general anesthesia [3,8,9]. Benzodiazepines typically exert less MEP suppression than agents such as propofol or thiopental, but continuous infusion has traditionally been limited due to prolonged recovery times and accumulation concerns.

Currently, limited clinical data are available on the effect of remimazolam on MEPs, particularly in a direct comparison with propofol. We therefore conducted a randomized crossover trial to compare the effects of remimazolam and propofol, both in combination with remifentanil, on intraoperative MEP amplitude and latency during spinal surgery. We hypothesized that remimazolam would have comparable or less suppressive effects on MEPs than propofol.

## 2. Materials and Methods

This crossover randomized controlled trial was approved by the Korea University Hospital Institutional Review Board (2022GR0051) on 8 February 2022 and was registered at ClinicalTrials.gov (NCT05453955) before the enrolment of the first patient. It was conducted between February 2022 and January 2023, and was performed in accordance with the Good Clinical Practice guidelines and the Declaration of Helsinki, and adhered to the guidelines of the Consolidated Standards of Reporting Trials (CONSORT) [10].

### 2.1. Patients

Patients aged 20–70 years who were scheduled to undergo elective spinal surgery requiring MEP monitoring were screened for eligibility. Patients were enrolled in this study after obtaining written informed consent. The exclusion criteria were as follows: an American Society of Anesthesiologists (ASA) physical status ≥ IV, a body mass index ≥ 40 kg m^2^, refusal by the patient to participate in the study, changes in the operative plan such as delay or cancelation of the operative schedule or cancelation of neuromonitoring, history of hypersensitivity to anesthetic agents, use of cardiac pacemakers, pre-existing neuromuscular disease, pre-existing motor weakness, and baseline neurological deficits.

Patients were randomly assigned to either the propofol first sequence or the remimazolam first sequence using a computer-generated randomization sequence with a 1:1 ratio, depending on the order in which the patients received propofol or remimazolam as the first agent. In the propofol first sequence, propofol was used for anesthesia induction (first agent), which was then switched to remimazolam (second agent) 60 min after the initial surgical incision. Conversely, in the remimazolam first sequence, remimazolam was used for anesthesia induction (first agent), which was then switched to propofol (second agent) 60 min after the initial surgical incision. The randomization sequence was generated using a computer-generated method.

### 2.2. General Anesthesia and Intervention Protocol

Upon entering the operating room, patients’ statuses were monitored using pulse oximetry, noninvasive blood pressure monitoring, electrocardiography, end-tidal carbon dioxide monitoring, and temperature measurement. The depth of anesthesia was monitored using the bispectral index (BIS; Coviden, Mansfield, MA, USA), and the degree of neuromuscular blockade was quantified using a TwitchView electromyograph (Blink Device Company, Seattle, WA, USA). After preoxygenation, general anesthesia was induced using either propofol (Fresofol MCT 2%, Fresenius Kabi, Homburg, Germany) or remimazolam (ByFavo, Hana Pharmaceutical, Seoul, Republic of Korea), combined with remifentanil (Ultiva, GlaxoSmithKline, Brentford, Middlesex, UK), according to the study’s protocols of allocation. In the propofol first sequence, both propofol and remifentanil were administered via target-controlled infusion (TCI), with a target effect-site concentration of 3–5 μg/mL and 3.0 ng/mL, respectively. In the remimazolam first sequence, remimazolam was continuously infused at a rate of 6–12 mg/kg/h for induction and then adjusted to 1–2 mg/kg/h for maintenance, with remifentanil similarly maintained via TCI.

Unlike propofol, remimazolam infusion was not guided by a pharmacokinetic model or effect-site targeting during the study period, as a clinically validated TCI model was unavailable at the time of study design. Instead, the infusion rate was titrated to maintain a BIS value of 40–60, in accordance with clinical practice guidelines. Although BIS monitoring was used to approximate equivalent anesthetic depth between agents, subtle pharmacodynamic differences cannot be excluded.

After loss of consciousness, 0.6 mg/kg of rocuronium was administered to both groups. Patients were intubated once a train-of-four (TOF) count of 0 was confirmed at the adductor pollicis muscle and BIS was below 60. After endotracheal intubation, the rate of remimazolam administration was adjusted to 1–2 mg/kg/h while maintaining the BIS at 40–60.

After positioning the patients for surgery, 2 or 4 mg/kg of sugammadex was injected according to the TOF count to achieve complete reversal of the neuromuscular blockade [11,12]. Consequently, the baseline MEP, defined as the MEP value for the first agent, was measured and recorded by a specialized nurse six times consecutively, at 5 min-intervals. Sixty minutes after performing the initial incision, the first agent was switched to the second agent. The crossover MEP, defined as the MEP value for the second agent, was measured six times consecutively, at 5 min-intervals, after at least 60 min of washout period. The second agent was maintained throughout the rest of the surgery, targeting a BIS of 40–60.

After completion of the surgery, the anesthetic drug infusion was ceased. If the BIS values remained below 60 for 10 min following the cessation of infusion, 0.2–0.5 mg of flumazenil was administered at the discretion of the anesthesiologist. The endotracheal tube was removed after confirmation of spontaneous breathing and response to verbal instructions. Patients were transferred to the recovery room following extubation.

### 2.3. Study Outcomes

The primary outcomes of this study were the MEP amplitude and latency. MEP data were recorded from the bilateral deltoids, abductor pollicis brevis, tibialis anterior, and abductor hallucis muscles using transcranial electrical stimulation with six square-wave stimuli (intensity, 200–400 mV; pulse duration, 200 µs; interstimulus interval, 200 µs). Interhemispheric stimulation was performed using subdermal needle electrodes placed at the C3 and C4 locations, according to the international 10–20 electroencephalogram (EEG) system. Montages at C3 and C4 were used to record MEP of the right and left extremities, respectively. The amplitude in our study refers to the peak-to-peak amplitude, and the latency refers to the onset latency [13].

The average values of the six measurements for each of the baseline and the crossover MEP were calculated for each patient. The other outcomes were the BIS value, blood pressure, heart rate, and TOF ratio at the time of measurement. Additionally, data on demographic characteristics, type of operation, duration of surgery and anesthesia, and the presence of recall were collected.

### 2.4. Statistical Analysis

Since no previous studies were available on the effect of remimazolam on MEP (when this research was initiated), the sample size was determined based on data from a previous crossover trial on the effects of nitrous oxide and propofol on MEP [14]. According to the mentioned study, MEP amplitude was 1031 (410) μV with nitrous oxide and 655 (466) μV with propofol. With a calculated effect size of 0.8532, statistical significance set at *p* = 0.05, and power of 0.9, a sample size of 18 patients was determined after performing the Wilcoxon signed-rank test. Accounting for a 10% dropout rate, we planned to recruit 20 patients.

Continuous data were presented as the mean with standard deviation (SD), or as the median with interquartile range [IQR]. Normality was confirmed using the Shapiro–Wilk test. Categorical data were presented as percentages with counts, analyzed using the Chi-square test or Fisher’s exact test. Paired *t*-test and Wilcoxon signed-rank test were used to compare the amplitude and latency of each anesthetic infusion. Statistical analyses were performed using the SPSS software (ver. 25.0; IBM Corp., Armonk, NY, USA).

## 3. Results

**Figure 1:**
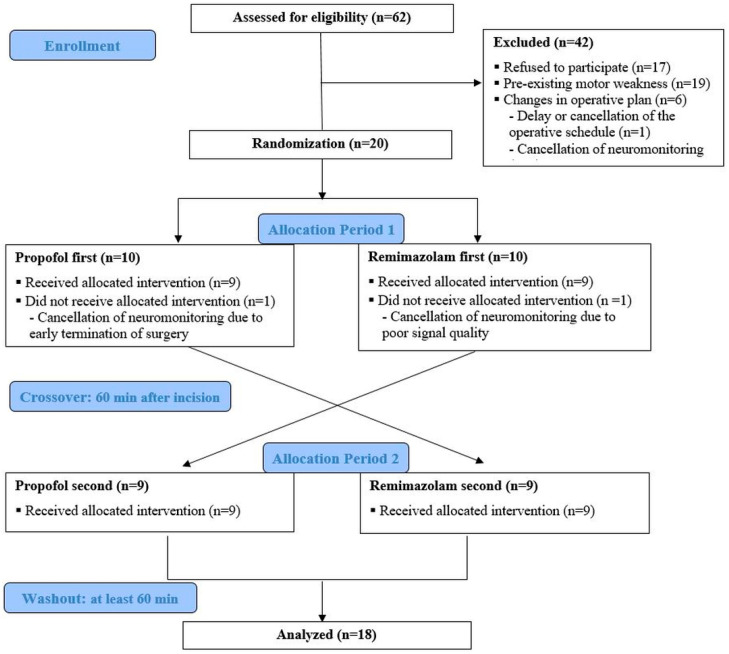
A flow diagram of the crossover study. Baseline MEPs were measured under the first anesthetic agent. Crossover to the second agent occurred 60 min after incision. A minimum washout of 60 min was ensured before the final MEP evaluation under the second agent.

Figure 1 demonstrates the CONSORT flow diagram used in this study. Of the 62 patients assessed for eligibility, 42 were excluded. Subsequently, 20 patients were randomly assigned to the propofol first (*n* = 10) and remimazolam first (*n* = 10) sequences. During the intervention, two patients (one from each assignment) were excluded due to canceled MEP monitoring. Ultimately, a total of 18 patients were included in the study.

No significant differences were observed between the propofol first and remimazolam first sequences regarding demographic and surgical data, including the duration of surgery and anesthesia, infused dose of anesthetics, emergence time, and length of hospital stay (Table 1).

**Figure 2:**
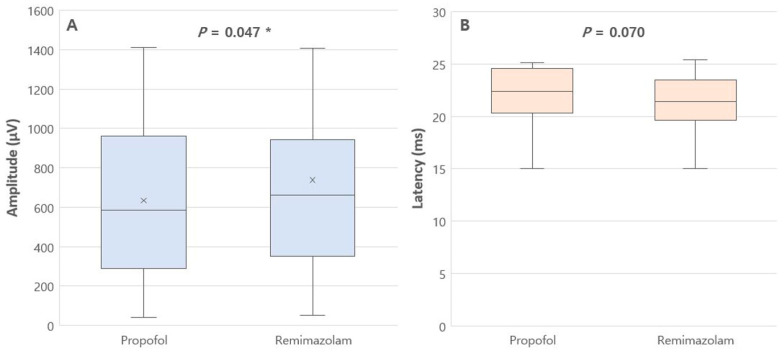
A box and whisker plot of MEP amplitude (**A**) and latency (**B**) during each anesthetic infusion. Horizontal bars represent the maximum, upper quartile (Q3), median, lower quartile (Q1), and minimum values. The “x” in the amplitude box indicates the mean value. *p*-Values for amplitude were obtained using the paired t-test, whereas *p*-values for latency were derived using the Wilcoxon signed-rank sum test. * *p* < 0.05, compared between the two anesthetic agents.

The MEP amplitude and latency during propofol infusion and remimazolam infusion were as follows: MEP amplitude was 635.3 (399.1) μV vs. 738.4 (480.4) μV, *p* = 0.047, and MEP latency was 22.4 [20.3–24.6] ms vs. 21.4 [19.6–23.5] ms, *p* = 0.070, respectively (Figure 2 and Table 2). These findings indicate that propofol causes significantly greater depression in MEP amplitude compared to remimazolam.

No significant differences were observed in blood pressure, heart rate, and TOF ratio between propofol infusion and remimazolam infusion. However, the median BIS value was higher for remimazolam infusion compared to propofol infusion (51.17 [42.86–56.83] vs. 41.28 [39.69–41.97]), although BIS values were maintained between 40 and 60 for all participants throughout the surgery (Table 2).

Two patients required an additional 5 mg of rocuronium administration during remimazolam infusion due to intraoperative movement. An incident of severe body movement disrupting surgery at the beginning of the operation occurred under remimazolam anesthesia at a dose of 2 mg/kg/h in a young, healthy, muscular male patient, although BIS values remained below 60. After the administration of 5 mg of rocuronium, the TOF ratio temporarily decreased to 73%, and the surgery was safely performed without interference with MEP monitoring. During and after surgery, no postoperative motor weakness or new neurological deficits were observed in any of the 18 patients.

## 4. Discussion

In this randomized crossover trial, we found that remimazolam, when used in combination with remifentanil, produced comparable or less suppression of MEPs than propofol during spinal surgery. Specifically, MEP amplitude was significantly better preserved with remimazolam, while latency remained similar. These findings suggest that remimazolam is a feasible alternative to propofol for anesthetic management in surgeries requiring intraoperative neurophysiological monitoring. In our study, the mean amplitude difference between propofol and remimazolam was approximately 100 μV, representing a modest absolute change but a meaningful proportional difference given the average amplitudes involved (approximately 635 μV vs. 738 μV). While this difference may not reach clinical warning thresholds [6], it suggests that remimazolam may help preserve MEP signal quality, which can be particularly important in borderline cases or challenging monitoring conditions.

Our results are consistent with the known pharmacodynamic profiles of these agents. Propofol, while widely used due to its titratability and rapid offset, is associated with dose-dependent suppression of cortical and spinal excitability, leading to reduced MEP amplitude [15,16]. Benzodiazepines such as midazolam have been shown to cause less MEP suppression, but their long duration of action previously limited their use in TIVA [17]. Remimazolam, a novel ultra-short-acting benzodiazepine, overcomes this limitation through organ-independent metabolism by carboxylesterase-1, and offers stable pharmacokinetics suitable for continuous infusion [9].

In our study, flumazenil was used in six patients in the propofol first sequence and one patient in the remimazolam first sequence, with no significant difference in the emergence time between the sequence assignments. The frequency of use in the propofol first was from long-term remimazolam was administered as the second agent (the first agent was infused only during the initial 60 min of surgery, after which the second agent was administered and maintained until the end of the procedure). In addition, remimazolam is known to maintain better hemodynamic stability compared to propofol [18]; however, no differences in hemodynamic variables were observed in our study. The lack of differences in the emergence times and hemodynamic variables in our study might be due to the relatively young age of the patients, with a mean age of 40 years. Additionally, the subjects were relatively healthy, with an ASA class of 1–2. Therefore, it is speculated that the hemodynamic deterioration effect and the delayed recovery of propofol were minimized in this study.

Although our findings support the use of remimazolam in neuromonitoring, some important clinical caveats emerged. In our study, intraoperative movement was observed in two patients during remimazolam infusion, despite BIS values being maintained within the 40–60 target range. In one case involving a young, muscular male, severe movement at the beginning of surgery disrupted the procedure and required rocuronium supplementation. This suggests that hypnotic levels based on BIS may not reliably correlate with adequate akinesia when using remimazolam. Although both anesthetics were administered to maintain a BIS target range of 40–60, the median BIS values were higher during remimazolam infusion. This may reflect relatively lighter anesthetic depth or altered/different EEG characteristics unique to remimazolam.

Notably, previous studies have shown that remimazolam tends to produce higher BIS and electromyographic values compared to propofol at equivalent sedation levels [19,20,21,22]. This could reflect a weaker suppressive effect on both cortical and neuromuscular activity, potentially leading to increased muscle tone or movement under conditions where neuromuscular blockade is not applied. Our observation aligns with this interpretation and implies that remimazolam may exert less suppression on electromyographic activity as well, despite similar EEG-derived indices.

This has important implications in clinical settings where complete neuromuscular blockade is avoided to enable MEP monitoring. While we ensured TOF ratios above 90% using sugammadex prior to MEP measurement, this does not reflect typical practice, where partial blockade is common [3]. Literature suggests that maintaining a TOF ratio of 20–50% can optimize surgical conditions without compromising MEP signal reliability [23,24,25]. Future studies should investigate the interaction between remimazolam and varying degrees of neuromuscular blockade on both MEP quality and movement control. Until these effects are better understood, careful intraoperative neuromuscular monitoring and individualized adjustment may be necessary when using remimazolam in surgeries requiring MEP monitoring.

To date, only a few case reports have been published on the effect of remimazolam on MEP monitoring [26,27], including only one controlled trial involving cervical spinal surgery [28]. In one report, remimazolam did not significantly decrease MEP amplitude, allowing adequate monitoring [26]. In another report, there was a decrease in MEP amplitude; however, it was speculated to be caused by a motor pathway abnormality rather than by remimazolam-induced anesthetic fade [27]. A recent randomized controlled trial investigated the effects of remimazolam and propofol on MEP changes (%) and found no significant differences between the two agents [28]. Our study differs in that we compared the magnitude of the amplitude (μV) of the effects of both drugs in a crossover fashion in the same patients, and established that less reduction in amplitude is associated with remimazolam. Further studies are required to elucidate the effects of remimazolam on MEP.

This study was conducted using a randomized crossover design. This was due to the inter-individual variability in MEP signal characteristics [29], and following consultation with an MEP engineer at our institution. It was decided that comparing the two drugs with very short half-lives in the same individual using a crossover design would be an appropriate approach. From one aspect, the randomized crossover design is superior to a randomized controlled design as it is less biased by individual differences [19]. A key strength of our study is the crossover design, which allows intra-patient comparisons and reduces interindividual variability in MEP responsiveness. Given the known variability in MEP signal amplitudes among patients, this approach enhances the internal validity of our findings. Furthermore, our study directly compared absolute MEP amplitudes (in µV) rather than percentage changes, providing more concrete clinical insight.

Nevertheless, several limitations must be acknowledged. First, the sample size was relatively small. However, our study was adequately powered based on a priori calculations using comparable crossover data [14]. Another recent crossover study comparing the effects of remimazolam and propofol on EEG also had a small sample size of 17 patients [19]. Furthermore, as the feasibility of remimazolam use in neuromonitoring is still under investigation, the present findings may serve as preliminary data to guide future studies in this field. Further studies with larger sample sizes will clarify these results. Second, although crossover designs are robust, there remains the possibility of residual anesthetic effects. To mitigate this, we ensured a washout period of at least 60 min before measuring crossover MEPs, which exceeds the context-sensitive half-times of both agents (typically <20 min for propofol and <10 min for remimazolam) [30,31,32,33,34,35]. (In our study, the crossover MEP was measured at the end of the surgical procedure, approximately 1–2 h after crossover). Third, no preoperative MEP data were available. Since the purpose of the study was a relative comparison of the two drugs, and considering the pain associated with MEP stimulation while awake, the MEP data before anesthesia were not measured. Fourth, although propofol was administered using a TCI system based on a pharmacokinetic model to maintain a stable effect-site concentration, remimazolam was administered using fixed-rate continuous infusion (mg/kg/h) without individualized pharmacokinetic guidance, as in 2022, when our study began, there was a lack of information on TCI models for remimazolam. This raises uncertainty as to whether anesthetic depth was consistently maintained within an optimal range for MEP monitoring during remimazolam anesthesia. According to the Masui model for remimazolam pharmacokinetics [36], infusion rates of 6–12 mg/kg/h for induction and 1–2 mg/kg/h for maintenance may result in significantly different plasma and effect-site concentrations depending on patient age and physiological variability. Incorporation of validated pharmacokinetic models in future studies could improve anesthetic precision and standardize conditions for neuromonitoring. Future studies may confirm this using pharmacokinetic-guided titration.

## 5. Conclusions

Remimazolam demonstrated comparable or superior effects to propofol on the MEP latency and amplitude when combined with remifentanil in spinal surgery. Remimazolam can be an effective alternative to propofol for MEP monitoring. Further research is needed regarding the use of remimazolam in neuromonitoring, especially in terms of its neuromuscular blockade effects.

## Figures and Tables

**Table 1:** Demographic and clinical data.

	Total Enrolled (*n* = 18)	Propofol First Assignment (*n* = 9)	Remimazolam First Assignment (*n* = 9)	*p*-Value
Age (yr)	40.50 (15.4)	38.8 (18.2)	42.2 (12.8)	0.066
ASA (I/II/III)	10/7/1	6/2/1	4/5/0	0.261
Height (m)	168.5 (5.4)	168.7 (5.3)	168.3 (5.7)	0.796
Weight (kg)	65.0 (14.0)	61.2 (12.7)	68.7 (15.0)	0.556
Duration of surgery (min)	242.8 (79.4)	246.1 (95.5)	242.4 (65.4)	0.435
Duration of anesthesia (min)	347.7 (107.2)	363.8 (133.4)	332.6 (78.1)	0.238
Monitoring site (APB/AH/others)	10/7/1	6/2/1	4/5/0	0.261
Propofol infused (mg)	1178 [925–1798]	1000 [720–1272]	1660 [1090–1927]	0.136
Remimazolam infused (mg)	201 [138–300]	300 [146–360]	178 [112–213]	0.063
Remifentanil infused (µg)	2399.7 (199.6)	2232.9 (837.9)	2354.4 (622.2)	0.299
Emergence time (min)	22.5 [11.5–37.5]	25.0 [16.0–50.0]	20.0 [10.0–32.5]	0.340
Flumazenil use (n)	7	6	1	0.050
Length of hospital stay (day)	7.0 [7.0–9.0]	7.0 [7.0–9.0]	7.0 [7.0–8.0]	1.000

Values are presented as mean (SD), median [IQR], or number of patients. ASA: American Society of Anesthesiologists physical status classification; APB: Abductor pollicis brevis; AH: Abductor hallucis; others: other monitoring sites, including the deltoid, biceps, and triceps.

**Table 2:** Perioperative data during propofol or remimazolam infusion.

	Propofol Infusion	Remimazolam Infusion	*p*-Value
MEP data			
Amplitude (μV)	635.3 (399.1)	738.4 (480.4)	0.047 *
Latency (ms)	22.4 [20.3–24.6]	21.4 [19.6–23.5]	0.070
Hemodynamic data			
Mean blood pressure (mmHg)	79.36 [74.06–84.51]	77.33 [73.80–82.30]	0.378
Heart rate (beats/min)	68.39 [59.67–75.89]	70.56 [66.94–72.19]	>0.999
Other data			
BIS value (0–100)	41.28 [39.69–41.97]	51.17 [42.86–56.83]	0.004 *
TOF ratio (%)	99.25 [97.97–100.44]	99.76 [99.11–100.85]	0.128

Values are presented as mean (SD) or median [IQR]. Average of six consecutive measurements at 5 min intervals when administering the two drugs to 18 patients. * *p* < 0.05, compared between the two anesthetic agents. MEP, motor-evoked potential; BIS, bispectral index; TOF, training of four.

## Data Availability

The data presented in this study are not publicly available due to institutional privacy policies and ethical restrictions, but may be made available from the corresponding author upon reasonable request and with appropriate institutional approval.

## Abbreviations

The following abbreviations are used in this manuscript:

TIVA	Total intravenous anesthesia
MEP	Motor evoked potential
BIS	Bispectral index
TCI	Target-controlled infusion
TOF	Train-of-four
EEG	Electroencephalogram
